# Grazing camels under semi-extensive production system: selectivity, feed intake capacity, digestion and energy expenditure

**DOI:** 10.1186/s12917-024-04199-1

**Published:** 2024-08-13

**Authors:** Ahmed R. Askar, Abdallah Masoud, Nasr E. El-Bordeny, Khalid Z. Kewan, Etab R. I. Abd El-Galil, Samir S. Abou El Ezz, Mohsen M. Shoukry

**Affiliations:** 1 Animal and Poultry Nutrition Department, Desert Research Center, Cairo, Egypt; 2 Animal Production Department, Faculty of Agriculture, Ain Shams University, Cairo, Egypt; 3 Animal and Poultry Physiology Department, Desert Research Center, Cairo, Egypt; 4 Animal Production Department, National Research Center, Cairo, Egypt

**Keywords:** Grazing camels, Plant selectivity, Digestibility, Heart rate, Energy utilization

## Abstract

**Background:**

It was proposed that camels are more effective than other livestock species in selecting plants for their nutritional value. They may self-regulate their voluntary feed intake to satisfy their nutritional needs. This study was designed to investigate camels’ feeding selectivity and ability to cover nutritional requirements when grazing alfalfa (high in protein) and/ or barley (high in energy) in a desert climate.

**Methods:**

Eighteen lactating camels were equally divided into three feeding treatments. They grazed daily on alfalfa, barley, or a mixed pasture of both, for two periods of one month each. The concentrate supplement was individually administered at 40 g/kg BW^0.75^, divided into two equal parts, in the morning and in afternoon. Total energy expenditure (EE) was estimated by heart rate (HR) monitors for 48 h after being calibrated by oxygen consumption using an upgraded face mask open-circuit respiratory system.

**Results:**

During the first period, camels had a greater forage intake and digestibility when they grazed barley rather than alfalfa, while those grazing mixed pasture performed intermediately. In the second period, camels had a similar forage intake and digestibility among treatments due to a decline in barley intake and digestibility compared to the first period, which was expected since the preferred plant part gradually shifted from barley grains to predominantly straw as a function of time. Similar HR and EE were found across periods and treatments. As a result of greater gross and digestible energy intake in period 1, a better energy balance in period 1 was observed compared to period 2. Camels better utilize barley than alfalfa. Grazing on barley had a higher energy balance than grazing alfalfa alone or in combination with barley. However, camels grazing barley produced lower milk yield and energy than those grazing alfalfa alone or in combination with barley, with no interaction detected between period and treatment.

**Conclusions:**

Lactating camels are able to self-regulate their voluntary intake to cover their energy requirements when they are grazing barley and/or alfalfa supplemented with a concentrate supplement at 40 g/kg BW^0.75^. Grazing barley is better utilized by camels than alfalfa. The chemical and physical properties of plant species play an important role in the selectivity of foraging camels. It also impacts their intake and digestibility, which is negatively associated with the proportion of cell wall content consumed.

## Introduction

In the face of climate change, camels are emerging as a crucial asset for food security. Their unique ability to utilize fibrous materials, which have limited nutritional value for humans directly, allows them to thrive in harsh environments and contribute to food production where other livestock may struggle. Camels are well-adapted to arid environments, desertification, and scare natural resources, and considered the most productive livestock species for milk and meat under these harsh conditions [1]. Although natural vegetation is relatively low and scattered in arid and semi-arid regions, the majority of camels rely on grazing on natural rangelands to meet their daily nutritional needs which may be considered the least expensive source of nutrients [2].

However, despite the larger body mass of camels, they consume less feed than other animal species [3, 4]. Hashi et al. [5] found that even with a ration containing 50% concentrate; camels dry matter intake was voluntary limited to 1.6–1.7% of body weight. Furthermore, feed intake capacity has been reported to be limited by roughage intake. Farid et al. [6] concluded that roughage intake in growing camels was limited in cafeteria diet (ad-lib concentrates and roughages) to be 16.1–33.4 g/ kg BW^0.75^. This wide variation in intake was attributed to the type of roughage. The limited roughage intake was also confirmed by Rabee and Askar [7] with growing camel calves. In addition, camels have been reported to be superior to other livestock species in selecting plants/ feeds of better quality [8]. They may develop mechanisms or behaviors that allow them to recognize feeds on the basis of their nutritional values [9]. They are able to self-regulate their voluntary intake to cover their energy needs when having a free choice to select diets from various concentrates and roughages [6]. Voluntary intake is often regulated by reticulorumen capacity, chemo-static feedback, and digesta passage rate [10]. On the other hand, heart rate was successfully used as an indicator for dynamic response to camel’s physical activity [11]. The current study aimed to investigate camels’ feeding selectivity and ability to cover nutritional requirements when grazing alfalfa (high in protein) and/or barley (high in energy), both of which are salt-tolerant plants, supplemented with concentrate in a desert climate, taking into consideration the use of heart rate as an indicator of energy expenditure in grazing camels.

## Materials and methods

The experiment was carried out at the “National Campaign for the Promotion of Camel Productivity” farm, Ras Sudr Research Station, Desert Research Center, Egypt. The station is located 200 kms from Cairo, the capital of Egypt, at latitude 29,35,30 N and longitude 32,42,20 E, the western coastal road to South Sinai. It is considered a desert climate with almost no rainfall during the year. The average annual temperature is 22 °C and yearly rainfall is 19 mm [12].

### Animals and treatments

This study was conducted in accordance with the Local Experimental Animal Care and Welfare Committee and approved by the Institutional Ethics Committee affiliated with the Animal and Poultry Production Division, Desert Research Center, Egypt (approval No. 7,092,020).

Eighteen lactating dromedary camels, approximately 12 years old, with an average initial live body weight of 395.7 kg (SE = 6.56), were used in the present experiment to study the diet selectivity, feed intake, and total tract digestibility, as well as milk yield and composition under grazing conditions. They were randomly distributed in three groups of six. The experiment lasted for two periods of one month each. Two-way analyses of variance were employed to compare three grazing treatments over two periods. All she-camels were daily grazing on alfalfa (Treatment A), barley (Treatment B), or a mixed pasture of both (Treatment AB). Alfalfa was almost in the early bloom stage, whereas barley was in the harvested (vitreous grain) stage. The area of the available pasture is around 6 acres, two acres for each treatment, with a stocking rate of three lactating camels per acre. Camels grazed the pasture continuously daily from 10:00 h to 16:00 h. The mixed pasture allowed alfalfa and barley to be grazed simultaneously, with about one acre allocated to alfalfa and another to barley. The concentrate supplement was individually administrated at 40 g/ kg BW^0.75^, divided into two parts, in the morning and in afternoon. Water was made available twice a day, right after the concentrate supplement feeding time. The concentrate supplement consisted of 55% corn grain, 10% cottonseed meal, 15% wheat bran, 15% soybean meal, 2.5% limestone, 1.5% salt, 0.5% sodium bicarbonate, 0.1% yeast, 0.1% antitoxin, and 0.3% minerals and vitamins premix (each 3 kg premix contains vitamin A: 10,000,000 iu, vitamin D3: 200,000 iu, vitamin E: 15,000 mg, manganese: 70 g, zinc: 60 g, iron: 50 g, copper: 15 g, iodine: 3 g, selenium: 0.3 g, and cobalt: 0.75 g). The chemical composition of concentrate supplement, and cultivated alfalfa and barley are shown in Table (1).

**Table 1:** Chemical composition of concentrate supplement, and cultivated barley and alfalfa based on a dry matter (DM) basis

Ingredients	Concentrate feed mixture	Barley			Alfalfa	
		Period			Period	
		1	2		1	2
Dry matter, g/ kg fresh matter	941	862	862		153	267
Gross energy, MJ/ kg dry matter	15.7	15.9	14.3		15.1	14.7
Chemical composition, g/ kg DM:						
Organic matter	909	890	836		861	885
Crude protein	158	115	77		160	130
Neutral detergent fiber	365	469	623		474	524
Acid detergent fiber	130	247	335		210	317
Acid detergent lignin	39.9	28.0	40.0		62.0	93.5

### Experimental procedures

All she-camels were chosen at the late-lactation period, at 8–9 months of lactation. They were individually hand-milked twice a day, morning and afternoon, in the presence of a suckling camel calve to stimulate milk secretion. The experiment lasted for two one-month periods, September and October, 2019. The last two weeks of each month were considered a measurement period which included one week of adaptation to harness and equipment, followed by another week of fecal collection and sampling. The animals were grazed daily between 10:00 h to 16:00 h. Each she-camel received the concentrate supplement twice a day, once in the morning before grazing (08:00 h) and once in the afternoon after grazing (16:00 h), while water was available throughout the day. The animals were weighed at the start and end of each period. During the collection period, fecal bags were emptied twice a day, at 06:00 h and 18:00 h. The total amount of feces was individually recorded daily. A sub-sample (10%) of each she-camel was taken and combined in individual composite samples. The hand-plucked simulated grazed samples were collected daily. The grazed and fecal samples were immediately air-dried at 65 °C for 48 h and kept for later analysis. The forage intake was estimated by an internal marker (acid insoluble ash, AIA [13]) based on the following equations:

1. The amount of internal marker in feces = The amount of internal marker in intake.
2. Fecal output * %internal marker in feces = Total intake * %internal marker in intake.
3. Total intake = (Fecal output * %internal marker in feces) / %internal marker in intake.

However, the digestibility (%) was calculated as follows:

4) Digestibility (%) = (Total intake – Fecal output) * 100/ Total intake.

Please note that the total intake equals the sum of the known concentrate supplement and estimated forage intakes. The pasture sample consists of hand-plucked simulated grazed samples of alfalfa only (Treatment A), barley only (Treatment B), or both (Treatment AB) based on the visual observation, assuming 100% fecal recovery of the internal marker.

### Energy expenditure

The calorimetry system and its usage were described previously by [14]. All animals were fitted with a face mask facilitating open-circuit respiration for measuring O_2_ consumption (Sable Systems, Las Vegas, NV). Heart rate (HR) was simultaneously measured to determine the individual energy expenditure (EE)/HR ratio. The concentration of O_2_ was determined with a fuel cell FC-1B O_2_ analyzer (Sable Systems, Las Vegas, NV), while EE was estimated by assuming a constant thermal equivalent of 20.47 kJ per liter O_2_ [15].

The human RCX3 monitors (Polar Electro Oy, Kempele, Finland) were considered to individually collect HR data at 1-min intervals for at least 48 h. The data was analyzed through the Polar Precision Performance SW software. The daily EE was estimated from the EE: HR ratio for each animal.

The gross energy (GE) of feed intake and feces were measured by bomb calorimeter (IKA, model C 200, Staufen, Germany), using benzoic acid as standard. Metabolizable energy (ME) was considered as 82% of digestible energy (DE) intake [16]. Energy balance was calculated as the difference between ME intake and total EE. The estimation of EE associated with grazing activity was based on estimates of total EE and ME intake, assuming an efficiency of ME utilization for maintenance of km at 0.66, where km = 0.35 (qm = ME/GE) + 0.503 [17].

### Weather data

A daily ambient temperature (T°C) and relative humidity (RH%) were obtained from Egyptian General Meteorological Authority. A temperature-humidity index (THI) was calculated with the following formula [18]:

THI = (0.8 × T) + [(RH / 100) × (T – 14.4)] + 46.4.

### Analytical procedures

Laboratory dry matter (DM) contents of feeds and feces were determined by drying at 105 °C for 24 h, and the organic matter (OM) was determined by ashing at 550 °C in a muffle furnace for 6 h. The crude protein (CP) was measured by the Kjeldahl method described in [19]. Neutral detergent fiber (NDF) content was determined in accordance with [20], and the acid detergent fiber (ADF) content was analyzed as described in [19] using the filter bag technique (ANKOM 200, ANKOM Technology Corp., Fairport, NY, USA). Acid detergent lignin (ADL, sa) content was determined according to [21]. The milk composition was determined using a milk scan instrument (Lactoscan S, Milkotronic LTD) after being stored at -20 °C immediately after milking.

### Statistical analysis

Data in Tables were statistically analyzed with the MIXED procedure of SAS [22]. Two-way analyses of variance were employed to compare three grazing treatments (plant species) over two periods and their interaction, with animal as the random effect. The model was Yijk = µ + Ti + Pj + (TP)_ij_ +eijk, where Yijk is the dependent variable, µ the overall mean, Ti the fixed treatment effect, Pj the fixed period effect, (TP)ij the fixed interaction effect of Ti and Pj and eijk is the residual. Both main effect and interaction means are presented regardless of significance of the interaction. Differences among means were determined by least significant difference with a protected F-test. In addition, hourly means for heart rate and energy expenditure were analyzed by the MIXED procedure of SAS, with hour a repeated measure and animal random and the subject. The model was Yijkl = µ + Ti + Pj + (TP)ij + Rijk + Hl + (TH)il + (PH)jl + (TPH)ijl + eijkl, where Yijkl is the dependent variable, µ the overall mean, Ti the fixed treatment effect, Pj the fixed period effect, (TP)ij the fixed interaction effect of Ti and Pj, Rijk the random effect of animal k in (TP)ij, Hl the fixed time effect (repeated measure), (TH)il the fixed interaction effect of Ti x Hl, (PH)jl the fixed interaction effect of Pj x Hl, (TPH)ijl the fixed interaction effect of Ti x Pj x Hl, and eijkl is the random error.

## Results

### Climate conditions

**Fig. 1:**
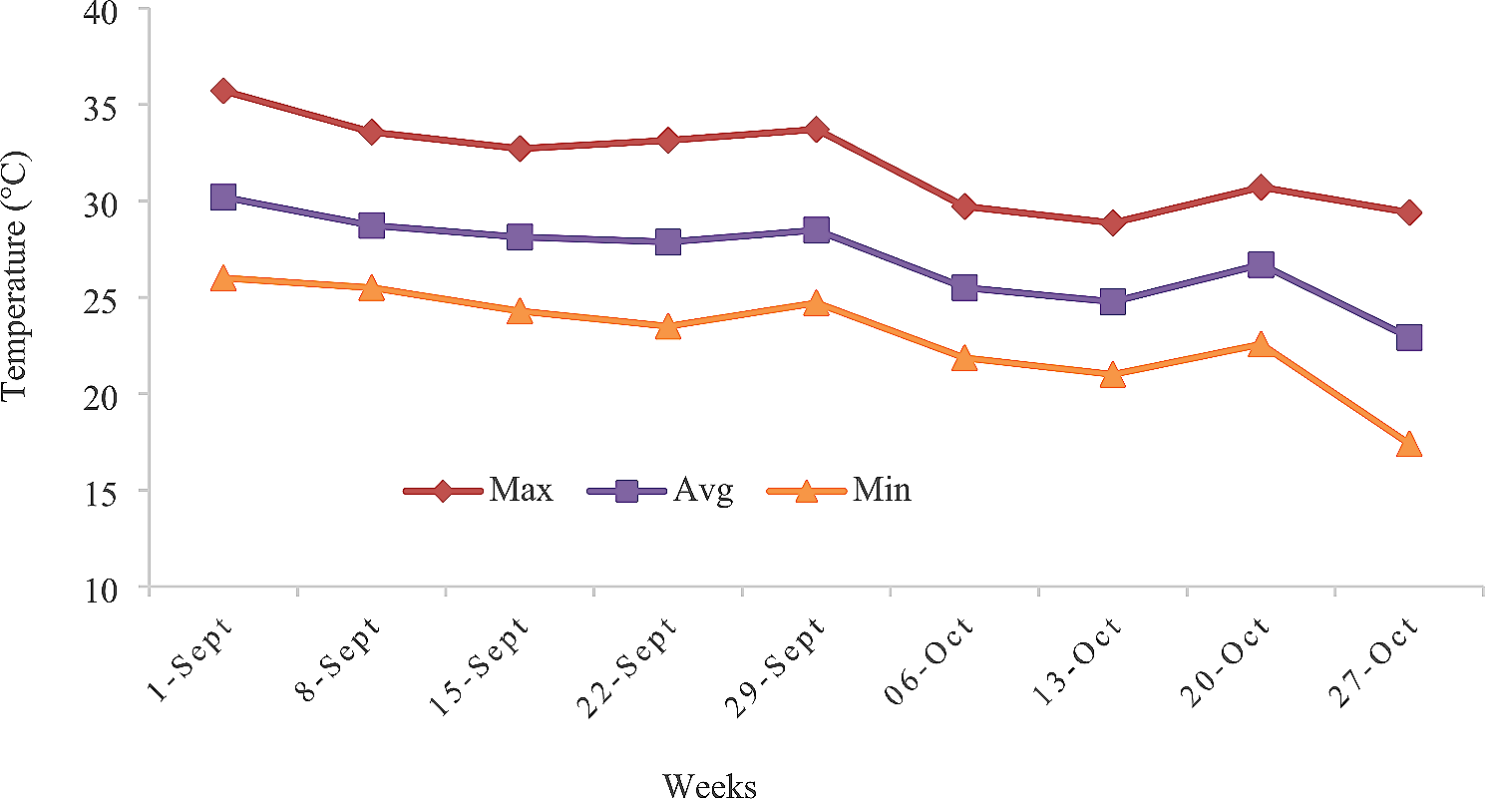
Mean, maximum and minimum temperature in 1-week periods throughout the experimental period that she-camels were exposed to September - October

**Fig. 2:**
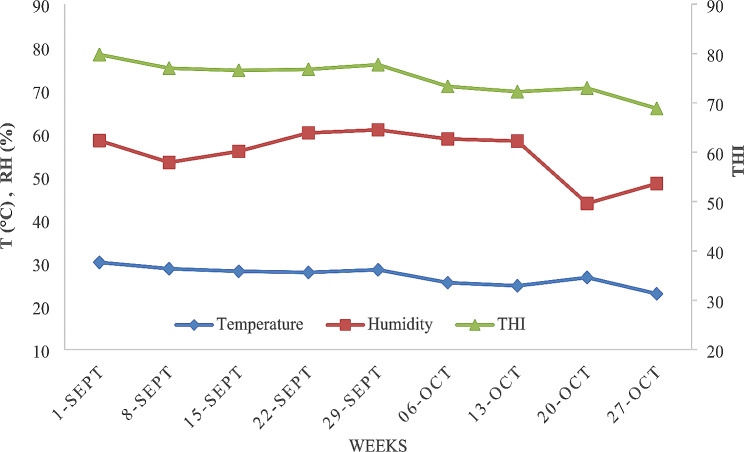
Mean temperature (T, °C), relative humidity (RH, %) and temperature–humidity index (THI) in 1-week periods throughout the experimental period that she-camels were exposed to September – October

The daily mean T, RH, and THI were obtained. The mean ambient temperature was 27.2 °C (Fig. 1), while the mean relative humidity and THI were 55.6% and 75.3%, respectively (Fig. 2).

### Intake and digestibility

A significant interaction (*P* < 0.001) indicated that forage intake (g/ kg BW^0.75^) was significantly (*P* < 0.001) greater for those grazing barley, compared to those grazing alfalfa alone or combined with barley during the first period, while a similar forage intake was observed among treatments during the second period (Table 2). As the concentrate supplement intake was similar (g/ kg BW^0.75^), total dry matter intake followed the same trend as the forage intake. However, the significant difference (*P* < 0.001) between periods, regarding the concentrate supplement intake, was due to the insignificant weight loss in the second period, as the same amount of concentrate was received by each animal during the two periods.

**Table 2:** Effect of period and plant species on feed intake by lactating camels grazing cultivated alfalfa, barley or a mixed pasture for both

Item		Period			Treatment				Period X Treatment							Significance		
				SEM				SEM	1			2			SEM			
		1	2		Alfalfa	Alfalfa-Barley	Barley		Alfalfa	Alfalfa-Barley	Barley	Alfalfa	Alfalfa-Barley	Barley		Period	Treat.	P*T
**Body weight**,** BW**																		
	Kg	402	401	7.3	399	400	405	8.98	401	401	404	397	399	405	12.7	ns	ns	ns
	kgBW^0.75^	89.8	89.5	1.22	89.3	89.4	90.2	1.50	89.6	89.6	90.1	89.0	89.2	90.2	2.11	ns	ns	ns
**Concentrate intake**,** DM / day**																		
	g/day	3736	3858	68.2	3787	3789	3815	83.6	3736	3737	3735	3838	3841	3895	118.2	ns	ns	ns
	g/kgBW^0.75^	41.6^b^	43.1^a^	0.19	42.4	42.3	42.3	0.23	41.7	41.7	41.4	43.1	43.0	43.1	0.32	***	ns	ns
**Forage intake**,** DM / day**																		
	g/day	1479	1408	61.9	1181^b^	1285^b^	1864^a^	75.9	963^c^	1200^bc^	2273^a^	1398^b^	1369^b^	1456^b^	107.3	ns	***	***
	g/kgBW^0.75^	16.4	15.7	0.57	13.2^b^	14.4^b^	20.7^a^	0.69	10.7^c^	13.4^bc^	25.1^a^	15.7^b^	15.3^b^	16.2^b^	0.98	ns	***	***
**Total intake**,** DM / day**																		
	g/day	5214	5266	115.7	4968^b^	5073^b^	5679^a^	141.7	4699^c^	4937^bc^	6007^a^	5236^bc^	5210^bc^	5351^b^	200.4	ns	**	*
	g/kgBW^0.75^	58.0	58.8	0.65	55.6^b^	56.7^b^	62.9^a^	0.79	52.4^c^	55.0^c^	66.6^a^	58.8^b^	58.4^b^	59.3^b^	1.12	ns	***	***

a, b and c Means without a common superscript letter in the row are differed (*P* < 0.05). ns = Non-significant; * = *P* < 0.05; ** = *P* < 0.01; *** = *P* < 0.001;

SEM = Standard error of means

The effects of grazing alfalfa, barley, or a mixed pasture of both, on nutrient intake and digestibility are showed in Table (3). The dry matter, organic matter, and crude protein digestibilities were greater (*P* < 0.01) for period 1, compared to period 2. The effect of plant species on dry matter digestibility was significant (*P* < 0.01) and followed the same trend of the dry matter intake, in which, a greater dry matter digestibility was observed for those grazing barley vs. alfalfa. The same trend was observed with the organic matter digestibility. On the contrary, grazing alfalfa alone, or in combination with barley, resulted in greater crude protein digestibility (*P* < 0.01) in comparison with grazing barley alone. However, the digestibility of fiber fraction (NDF) was increasing with the increase of barley in the diet. Camels grazing cultivated barley showed greater (*P* < 0.001) NDF digestibility compared to those grazing alfalfa, while those grazing mixed pasture had an intermediate value (Table 3).

**Table 3:** Effect of period and plant species on nutrients intake and digestibility by lactating camels grazing cultivated alfalfa, barley or a mixed pasture for both

Item		Period			Treatment				Period X Treatment							Significance			
				SEM				SEM	1			2			SEM				
		1	2		Alfalfa	Alfalfa-Barley	Barley		Alfalfa	Alfalfa-Barley	Barley	Alfalfa	Alfalfa-Barley	Barley		Period	Treat	P*T	
**Dry matter intake**,																			
	g/day	5214	5266	115.7	4968^b^	5073^b^	5679^a^	141.7	4699^c^	4937^bc^	6007^a^	5236^bc^	5210^bc^	5351^b^	200.4	ns	**	*	
	g/kg BW^0.75^	58.0	58.8	0.65	55.3^b^	56.7^b^	62.9^a^	0.79	52.4^c^	55.0^c^	66.6^a^	58.8^b^	58.4^b^	59.3^b^	1.12	ns	***	***	
	Digestion, %	59.4^a^	55.6^b^	0.58	55.4^b^	57.3^b^	59.7^a^	0.71	56.4^bc^	59.0^b^	62.8^a^	54.5^c^	55.7^c^	56.6^bc^	1.00	***	**	**t**	
**Organic matter intake**,																			
	g/day	4704	4718	103.6	4476^b^	4568^b^	5088^a^	126.9	4225^c^	4467^b^	5418^a^	4726^b^	4669^b^	4758^b^	179.5	ns	**	**	
	g/kg BW^0.75^	52.3	53.7	0.57	50.1^b^	51.0^b^	56.4^a^	0.70	47.1^d^	49.8^cd^	60.0^a^	53.1^b^	52.3^bc^	52.7^b^	0.99	ns	***	***	
	Digestion, %	65.0^a^	60.8^b^	0.48	61.0^c^	62.9^b^	64.9^a^	0.59	62.1 ^c^	64.8^b^	68.1^a^	59.9 ^c^	60.9 ^c^	61.7 ^c^	0.83	***	***	t	
**Crude protein intake**,																			
	g/day	910^a^	854^b^	18.5	883	847	915	22.6	876^b^	867^b^	986^a^	889^b^	827^b^	845^b^	32.0	*	ns	t	
	g/kg BW^0.75^	10.1^a^	9.5^b^	0.09	9.9^c^	9.5^b^	10.1^a^	0.11	9.7^b^	9.7^b^	10.9^a^	10.0^b^	9.3^c^	9.3^c^	0.16	***	**	***	
	Digestion, %	60.5^a^	55.9^b^	0.57	59.6^a^	58.7^a^	56.2^b^	0.70	61.5	61.4	58.5	57.7	56.0	53.9	0.99	***	**	ns	
**Neutral detergent fiber intake**,																			
	g/day	2227^b^	2412^a^	52.4	2157 ^b^	2213^b^	2589^a^	64.2	1988^c^	2077^c^	2616^a^	2325^b^	2348^b^	2563^ab^	90.8	*	***	t	
	g/kg BW^0.75^	24.8^b^	27.0^a^	0.32	24.1^b^	24.7^b^	28.7^a^	0.49	22.1^c^	23.1^c^	29.0^a^	26.1^b^	26.3^b^	28.4^a^	0.55	***	***	***	
	Digestion, %	51.3	51.4	0.56	46.2^c^	51.6^b^	56.3^a^	0.69	45.1	52.2	56.7	47.3	51.1	55.9	0.97	ns	***	ns	

a, b and c Means without a common superscript letter in the row are differed (*P* < 0.05). ns = Non-significant; t = 0.05 ˂ *P* < 0.10; * = *P* < 0.05; ** = *P* < 0.01; *** = *P* < 0.001; SEM = Standard error of means

On the other hand, camel milk yield was not affected by period but was considerably lower (*P* < 0.01) for those grazing barley vs. alfalfa. There were no significant differences in milk composition among treatments or periods (Table 4).

**Table 4:** Effect of period and plant species on milk yield and composition by lactating camels grazing cultivated alfalfa, barley, or a mixed pasture for both

Item		Period			Treatment				Period X Treatment							Significance		
				SEM				SEM	1			2			SEM			
		1	2		Alfalfa	Alfalfa-Barley	Barley		Alfalfa	Alfalfa-Barley	Barley	Alfalfa	Alfalfa-Barley	Barley		Period	Treat	P*T
**Yield**																		
	kg/day	2.71	2.39	0.17	2.87^a^	2.72^a^	2.07^b^	0.21	3.17	2.76	2.21	2.56	2.69	1.93	0.30	ns`	*	ns
**Composition**,** %**																		
	Fat	3.33	3.62	0.44	3.24	3.86	3.33	0.54	2.92	4.02	3.05	3.56	3.70	3.62	0.76	ns	ns	ns
	Lactose	3.91	4.28	0.16	4.05	4.18	4.05	0.20	3.95	4.00	3.80	4.16	4.37	4.30	0.28	ns	ns	ns
	Protein	2.60	2.83	0.11	2.69	2.77	2.68	0.13	2.62	2.65	2.52	2.76	2.90	2.84	0.18	ns	ns	ns

a, b and c Means without a common superscript letter in the row are differed (*P* < 0.05). ns = Non-significant; t = 0.05 ˂ *P* < 0.10; * = *P* < 0.05; ** = *P* < 0.01, *** = *P* < 0.001; SEM = Standard error of means

### Energy utilization

The effect of grazing alfalfa, barley, or a mix of both, on energy utilization are shown in Table (5). The significant interaction (*P* < 0.001) between period and treatment indicated that GE intake (kJ/ kg BW^0.75^) was significantly higher for those grazing barley in period 1, while a similar GE intake was observed among treatments in period 2. A similar pattern was observed for digestible and metabolizable energy intake (kJ/ kg BW^0.75^, Table 5). However, period 1 had significantly (*P* < 0.001) greater digestible energy (%) compared to period 2. Grazing barley resulted in higher (*P* < 0.01) digestible energy (%) compared to alfalfa alone or mixed with barley (Table 5).

**Table 5:** Effect of period and plant species on energy utilization by lactating camels grazing cultivated alfalfa, barley, or a mixed pasture for both

Item		Period				Treatment								Period X Treatment								Significance					
				SEM								SEM		1				2			SEM						
		1	2			Alfalfa		Alfalfa-Barley		Barley				Alfalfa		Alfalfa-Barley	Barley	Alfalfa	Alfalfa-Barley	Barley		Period		Treat		P*T	
**Gross energy**,** GE**																											
	MJ/day	79.6	80.4	1.71		77.2^b^		78.7^b^		85.0^a^		2.10		74.0^c^		76.0^c^	89.0^a^	80.3^b^	80.0^b^	81.3^b^	2.97	**ns**		*		*	
	kJ/BW^0.75^/day	885	899	9.2		864^b^		869^b^		942^a^		11.2		826^c^		846^c^	984^a^	902^b^	892^b^	901^b^	15.9	**ns**		***		***	
**Digestible energy**,** DE**																											
	MJ/day	46.1^a^	42.0^b^	0.86		41.2^b^		43.0^b^		48.3^a^		1.06		42.0^b^		44.0 ^b^	53.0^a^	41.0 ^b^	42.0 ^b^	44.0 ^b^	1.50	**		***		*	
	kJ/BW^0.75^/day	514.0^a^	469.5^b^	4.83		462.0^b^		477.1^b^		537.0^a^		5.92		467.0^b^		486.2 ^b^	589.0^a^	456.4 ^b^	468.0 ^b^	484.2 ^b^	8.37	***		***		***	
	%	57.9^a^	52.2^b^	0.46		53.5^b^		55.0^b^		57.0^a^		0.56		56.5		57.5	60.0	50.5	52.4	54.0	0.80	***		**		**ns**	
**Metabolizable energy**,** ME**																											
	MJ/day	37.8^a^	34.4^b^	0.71		34.0^b^		35.0^b^		40.0^a^		0.87		34.3^b^		36.0 ^b^	43.5^a^	33.2 ^b^	34.2 ^b^	36.0 ^b^	1.23	**		***		*	
	kJ/BW^0.75^/day	421.4^a^	385.0^b^	5.59		379.0^b^		391.2^b^		440.0^a^		6.84		383.0^b^		399.0^b^	483.0^a^	374.2^c^	384.0^b^	397.0^b^	9.69	***		***		***	
**Heart rate**,** HR**																											
	Beat/min	47.80	47.40	1.500		45.90		49.80		47.10		1.830		45.00		49.40	49.00	46.8	50.20	45.20	2.590	**ns**		**ns**		**ns**	
**Energy expenditure**,** EE**																											
	kJ/BW^0.75^/day	315	313	13.1		327		305		310		16.1		320		303	323	333	307	298	22.8	**ns**		**ns**		**ns**	
**Heat Increment**																											
	kJ/BW^0.75^/day	98.2	95.6	13.14		109.6		88.0		93.3		16.09		102.9		85.9	105.8	116.2	90.0	80.7	22.75	**ns**		**ns**		**ns**	
**Energy balance**,** EB**																											
	kJ/BW^0.75^/day	106.2^a^	72.3^b^	11.24		51.9^b^		86.2^b^		129.7^a^		13.76		63.0		95.7	160.1	41.0	77.0	99.3	19.47	*		**		**ns**	

a, b and c Means without a common superscript letter in the row are differed (*P* < 0.05); ns = Non-significant; t = 0.05 ˂ *P* < 0.10; * = *P* < 0.05; ** = *P* < 0.01; *** = *P* < 0.001; SEM = Standard error of means

**Fig. 3:**
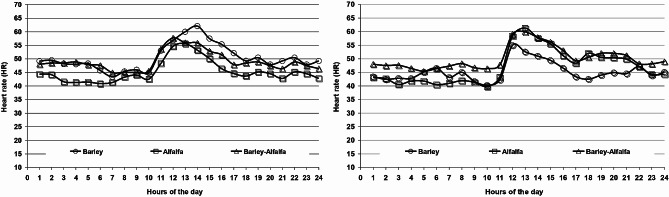
Effect of feeding barley, alfalfa or both on heart rate during the 1st and 2nd periods, respectively

**Fig. 4:**
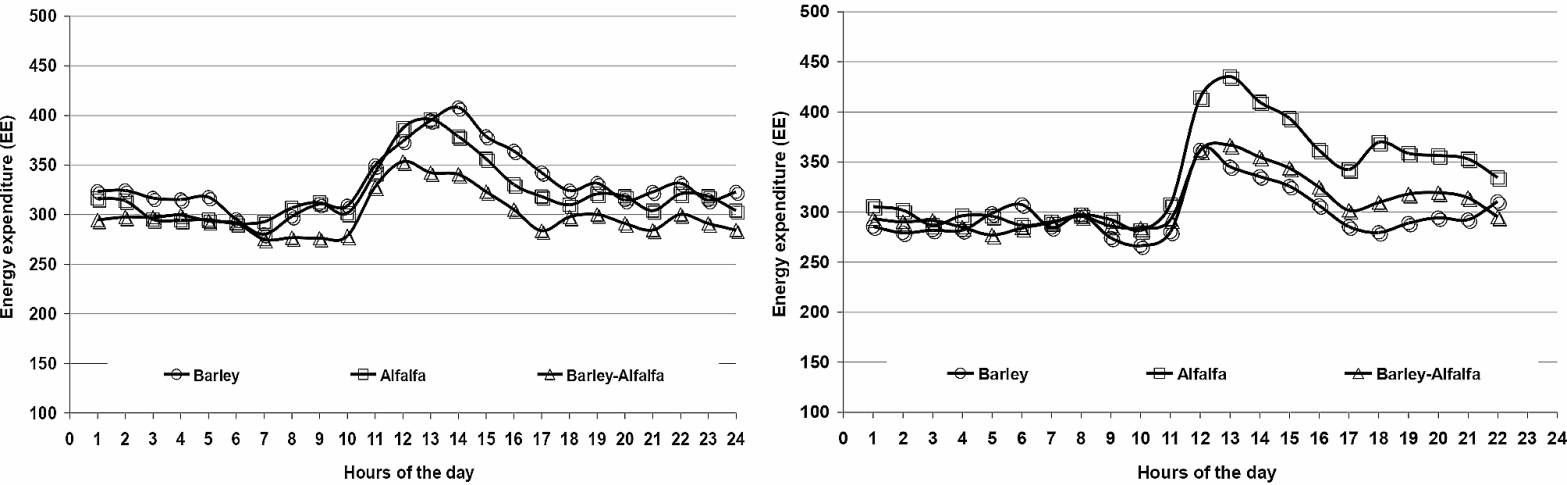
Effect of feeding Barley, alfalfa or both on energy expenditure during the 1st and 2nd periods, respectively

On the other hand, similar HR and EE were detected across periods and treatments (Table 5). As a result, increased GE and DE intake in period 1 resulting in a better energy balance (*P* < 0.05) compared to period 2. Similarity, grazing barley had a higher energy balance (*P* < 0.01) than those grazing alfalfa alone or combined with barley. Moreover, HR and the corresponding EE throughout 24 h of the day are illustrated in Figs. (3 and 4). During grazing time (10:00 h – 16:00 h), it is clear that EE was greater (*P* < 0.05) for those fed barley vs. alfalfa in the period 1, while the contrary was observed in period 2.

## Discussion

### Intake and digestibility

The extension of cultivated salt-tolerant plants to reduce the cost of feeding is one of the primary goals for the sustainable development of animal production in desert areas, particularly those affected by salinity, such as the current study area. Cultivated alfalfa is considered a perennial plant species, while barley is a annual one. However, in period 1, camels had a greater forage intake (g/ kg BW^0.75^) and digestibility (%) when they grazed barley rather than alfalfa (Tables 2 and 3). This might imply that grains were primarily chosen as a source of non-structural carbohydrates with high dry matter (86.2%) and energy (15.9 MJ/ kg DM) contents (Table 1), which led to increased dry and organic matter intake and digestibility (Table 3). Camels have been shown to be more effective than other livestock animals at selecting high-quality forage [8]. They may develop systems or habits that enable them to identify feed depending on its nutritional value [9]. When given the option of selecting from a variety of concentrates and/or roughages, they can self-regulate their voluntary intake to meet their energy requirements [6]. The same authors confirmed that this mechanism is influenced by the type or quality of roughage. On the contrary, Camels had a similar forage intake and digestibility among treatments in the second period, which was due to a decline in the intake and digestibility for those grazing barley compared to the first period. This was expected since the preferred plant part gradually shifted from barley grains (non-structural carbohydrates) to predominantly straw (high cell-wall content) as a function of time. The greater fiber fraction contents of barley in period 2, compared to period 1 (Table 1), supported the results. This may reflect the interaction between limited rumen fill capacity and rumen outflow rate. It is known that when NDF content goes up, intake capacity is reduced [23]. Chemical and physical characteristics of plant species were reported to be a major factor for selectivity by foraging camels on natural rangelands [24]. However, dry and organic matter digestibility was reported to be negatively associated with the proportion of cell-wall content consumed [25].

The fiber fraction digestibility was found to be greater for camels grazing barley vs. alfalfa, which may be due to the low lignin content in barley (Table 1), while those grazing a mixed pasture of both plant species had an intermediate value (Table 3). The partial replacement of alfalfa by barley (mixed pasture treatment) in the diet, together with a decrease in dry matter intake, may have negatively affected the rumen rate of passage, increased the retention time, and allowed more time for microbiota to efficiently digest fibrous materials. It was found that roughage source affected chewing time and, indirectly, the roughage index value [26], which expected that rumination time was higher for feeding straw instead of alfalfa. Increasing rumination time may enhance saliva production, giving more buffering capacity in rumen that could improve fiber digestibility condition [27]. On the other hand, as the availability of soluble carbohydrate and nonprotein nitrogen in alfalfa hay is higher than that in barley straw, greater rumen volatile fatty acids were expected to be produced when camels fed alfalfa hay vs. barley straw which negatively affected rumen pH that is not in favor of fiber digestibility [28] but may favor crude protein digestibility, as shown in Table (3).

On the other hand, feed intake capacity was limited by roughage intake that ranged from 10.7 to 25.1 g/ kg BW^0.75^ (Table 2). Farid et al. [6] found that roughage intake in growing camels was limited in cafeteria diet (*ad-lib* concentrates and roughages) to be 16.1–33.4 g/ kg BW^0.75^ that was similar to our findings and attributed this wide variation in intake to the type or quality of roughage. However, total dry matter intake in the current study ranges from 1.17 to 1.48% of body weight (52.4–66.6 g/ kg BW^0.75^, Table 2). The findings were consistent with [29] and [30] who estimated a camel’s dry matter intake to be approximately 55.1–62.9 g/ kg BW^0.75^. Despite the large size of camels, they consume less feed than other animal species [3, 4, 7]. Askar [14] and Askar et al. [31] reported that dry matter intake of 2% of body weight may approximately cover only the maintenance requirements of sheep or goats. Hashi et al. [5] stated that even with a ration containing 50% concentrate, dry matter intake of camels was voluntarily limited at 1.6–1.7% of body weight. Recently Rabee and Askar [7] and Askar et al. [32] confirmed those findings with growing camels.

Although a lower dry matter intake for those grazing alfalfa vs. barley, in the current study, milk yield was considerably higher when alfalfa vs. barley was fed which was probably associated with the higher crude protein intake and digestibility (Table 3). Dereje and Udén [33] found that milk yield significantly increased when lactating camels on range supplemented with high protein vs. high energy supplement. The author stated that increasing milk yield could increase calf survival rate and have a positive effect on overall herd productivity.

### Energy utilization

Heart rate has been successfully employed as an indicator for dynamic response for camels’ physical activity [11], which is reflected in the energy expenditure as a part of the energy cost for activity [34]. Furthermore, HR has been extensively applied as an indicator for the EE with sheep and goats [13, 31, 35], cattle [36], and camels in the current study (Table 5), by measuring oxygen consumption and HR simultaneously.

Although a greater grazing activity reported for those grazed barley vs. alfalfa during the grazing time in period 1 (Figs. 1 and 2), the relation between EE and ME intake shows that the camels fed barley are in a better state with a lower EE (EE = 66.9 and 83.6% of ME intake, respectively) and a higher energy balance than those fed alfalfa, while those that grazed on both have an intermediate value (Table 5). Findings are consistent with the greater intake, mainly forage, for those fed barley vs. alfalfa (Table 2). In this regard, a positive relationship between the level of feed intake and EE was established in camels [37], and in sheep and goats [13, 31, 38, 39]. However, a greater EE for those fed alfalfa during grazing time in period 2 suggests higher grazing activity searching for feed, in comparison with those fed barley straw, taking into consideration the high dry matter content of barley vs. alfalfa, and the fact that alfalfa is substantially less bulky than barley. It has been reported that the EE is also affected by the quality of the feed, with a lower EE observed when low-quality forage was given, such as *Acacia saligna* [40], wheat straw [41], or *Atriplex nummularia* [31], compared to alfalfa hay, that is mainly also related to a lower feed intake.

The HR and EE increased rapidly when the camels started grazing, and then decreased gradually until they stopped grazing (Figs. 1 and 2), demonstrating that camels quickly adapted to the new grazing conditions. Findings are in coherence with those of [11] when the load is released, indicating a moderately stressful condition.

Despite the low intake, lactating camels in the present study were satisfactorily maintained, particularly when they grazed on barley, which resulted in higher GE and DE intake (Table 5). When the net energy requirements for maintenance was estimated based on energy balance and the net energy supplied by diet (197, 164 and 172 kJ/kg BW^0.75^ for those fed alfalfa, barley, or both, respectively), assuming an efficiency of ME utilization for maintenance of km 0.66, where km = 0.35 (qm = ME/GE) + 0.503 [17], these values were about 20% lower than those published for fasting metabolic rate for camels (222 kJ/kg BW^0.75^, 37). However, the current estimated MEm for camels were around 300, 245, and 261 kJ/kg BW^0.75^, respectively, for those grazing on alfalfa, barley, or both, respectively, calculated on the basis of their energy balance (Table 5) and the net energy for maintenance, assuming a km of 0.66 [17]. The reported MEm, in the current study, are higher than those reported by [42] (217 kJ/ kg^0.75^) and is very close to those reported by [37] (305 KJ/kg BW^0.75^) in camels. On the contrary, this value was markedly below than those reported for sheep ( [43], 450 kJ/kg BW^0.75^; [44], 431 kJ/kg BW^0.75^) and goats ( [45], 429 kJ/kg BW^0.75^; [14], 398 kJ/kg BW^0.75^).

Camels were reported to be maintained on a low intake of low-quality forage (3–5 kg, [46]) which indicated that they could extract the most energy possible from fibrous materials. Several studies have concluded that camels’ nutrients requirements are lower than those of other ruminant species. Camels require about 30–40% less ME for maintenance (MEm) than sheep or goats (303 kJ/ kg BW^0.75^ for camels, [37] vs. 429–450 kJ/kg BW^0.75^ for sheep and goats, [31, 43–45]). These findings coincide with a low MEm reported for camels by [47] and [48]. In support, Macfarlane [49] reported a low metabolic rate of camels compared to that for sheep and cattle. Findings were recently confirmed by Dittmann et al. [50]. Moreover, because camels have a great capacity to recycle urea as a source of nitrogen when dietary protein is limited, they require less protein than other ruminant species. Camels appear to be more susceptible to energy deprivation than protein deficiency, and they can eat low-quality forage if exclusively forced to [7, 51].

Although a lower intake was noted for those grazing a mixed pasture of alfalfa and barley, compared to those grazing only barley in period 1, they were satisfactorily maintained (Table 2), suggesting that camels are efficient users of pasture. Wilson [52] indicated that camels usually do not browse on one individual plant species but they take a few bites and move to another. Foraging camels normally spread over a large area, thus minimizing pressure on a particular area and avoiding overgrazing [53]. Food scarcity is common in desert areas, particularly during the drought season [13]. As a result, animals living in these environments are expected to have some mechanism for survival. It has been found that in extreme cases of limited natural vegetation, camels not only reduce their feed intake, but also their metabolic rate [42, 54]. Desert animals, such as black Bedouin/ Balady goats [14, 45, 55] and Barki sheep [44, 56] have been reported to be able to reduce their basal metabolic rate as a survival mechanism when subjected to drought or feed shortage. This could be explained by a reduction in energy required by the splanchnic tissues [57] which account for a sizable component of the fasting metabolic expenditure [16]. A lower heat production was observed in desert sheep and goats when they consumed low quality forage, *Acacia saligna* [40], wheat straw [41], or *Atriplex nummularia* [31], compared to alfalfa hay, suggesting that desert animals can reduce their metabolic rate when consuming low quality forage. The findings agreed with those reported by [38] and [31, 44] who concluded that sheep and goats can markedly reduce their heat production when ME intake is restricted below maintenance requirement.

## Conclusions

Lactating camels are able to self-regulate their voluntary intake to cover their energy requirements when they are grazing barley and/or alfalfa supplemented with a concentrate supplement at 40 g/kg BW^0.75^. Grazing barley is better utilized by camels than alfalfa. Furthermore, because barley consumes less water than alfalfa, it is recommended for cultivation in desert areas as an alternate feed source. The chemical and physical properties of plant species play an important role in the selectivity of foraging camels. It also impacts their intake and digestibility, which is negatively associated with the proportion of cell wall content consumed.

## Data Availability

No datasets were generated or analysed during the current study.

## Abbreviations

BW: Body weight
DM: Dry matter
DMI: Dry matter intake
OM: Organic matter
CP: Crude protein
AIA: Acid insoluble ash
NDF: Neutral detergent fiber
ADF: Acid detergent fiber
ADL: Acid detergent lignin
GE: Gross energy
DE: Digestible energy
ME: Metabolizable energy
MEm: Metabolizable energy used for maintenance
EE: Energy expenditure
HR: Heart rate
Km: Efficiency of ME used for maintenance
MJ: Mega Joul
kJ: Kilo Joul
kg: Kilo gram
O2: Oxygen
RH: Relative humidity
T: Ambient temperature
THI: Temperature–humidity index
vs.: Versus
SEM: Standard error of means
